# Evolutionary game analysis of carbon emission reduction of Internet enterprises under multiple constraints

**DOI:** 10.1371/journal.pone.0296918

**Published:** 2024-01-31

**Authors:** Maochun Zhou, Lei Qian

**Affiliations:** School of Business Administration, Liaoning Technical University, Huludao City, Liaoning Province, China; Anhui Polytechnic University, CHINA

## Abstract

With the rapid development of information technology, internet enterprises have sprung up, bringing about huge power consumption due to constantly expanding enterprise scale, which in turn leads to significant carbon emissions. Additionally, the large influence of internet enterprises on the public and other businesses makes it particularly necessary to pay attention to their carbon emission reduction efforts. To explore the evolution path and patterns of carbon emission reduction among internet enterprises under the carbon neutrality goal, this paper constructs an evolutionary game model for internet enterprises to enter the carbon emissions trading market based on Externality and Sustainable Development Theories, while considering constraints from the carbon market, financial institutions and the public. The model utilizes Python 3.8.2 software for numerical simulations, aiming to push internet enterprises towards low-carbon development. The research findings indicate that: (1) Carbon emission reduction behavior of internet enterprises exhibits significant externality, and when constraints are weak or incentives are not evident, the motivation for enterprises to reduce carbon emissions is insufficient. (2) The carbon market can effectively promote carbon emission reduction among internet enterprises, and the strategy of entering the carbon market becomes the preferred option for these enterprises gradually. (3) Multiple constraints, including emission reduction costs, penalty for non-compliance, government subsidies, financing costs, opportunity losses, and reputation losses, can force internet enterprises towards low-carbon development.

## 1. Introduction

Climate warming and environmental protection have become common issues for all of humanity. In the new stage of development, "green and low-carbon" has become a crucial strategy for high-quality development in China. In September 2020, China officially proposed the goals of "peaking carbon emissions by 2030 and achieving carbon neutrality by 2060" at the 75th United Nations General Assembly. In July 2021, the national carbon emissions trading market was officially launched. The emergence of carbon trading increases the carbon emission costs for high-carbon enterprises while providing another channel for low-carbon enterprises to obtain economic benefits, which will force enterprises to take the initiative in emission reduction, thus achieving a win-win situation for the economy, environment, energy, and climate.

The rapid development of the digital economy has provided vast market opportunities for Internet enterprises. In December 2021, the State Council released the "14th Five-Year Plan and Long-Range Objectives Through the Year 2035" which clarified China’s strategic direction towards a comprehensive digital era. In October 2022, the report delivered at the 20th National Congress of the Communist Party of China emphasized the acceleration of building a powerful network country and digital China. In October 2023, the 3rd Belt and Road Initiative International Cooperation Forum positively evaluated the outstanding contributions of the "Digital Silk Road" in promoting the development of the digital economy in various countries. With the strong global development of the digital economy and the deepening of the "Digital Silk Road" initiative, internet enterprises have sprung up rapidly, continuously expanding in scale and number, becoming important driving forces for China’s march towards the digital era.

Internet enterprises possess vast amounts of data and employees, and their data centers, super computing centers, product supply chains, as well as administrative offices, all produce significant carbon emissions [1]. Compared to key emitting industries, although the current proportion of carbon emissions from internet enterprises may be relatively small, the adjustment of industrial structures is expected to result in a considerable impact from their carbon footprint on the overall emissions landscape. Additionally, given the widespread influence of internet enterprises, their strong exemplary and driving roles in low-carbon emission reduction demonstrate a notable guiding effect on both the public and other enterprises. Therefore, exploring effective pathways for internet enterprises to achieve low-carbon emissions within the context of carbon trading represents a compelling topic worthy of further examination.

## 2. Literature review

### 2.1 Research on carbon emission reduction effect of carbon trading

Carbon trading, as a significant strategy to reduce greenhouse gas emissions and address climate change, has gained widespread recognition from the international community. The ultimate goal of carbon trading is not merely the transaction itself, but rather the achievement of carbon emission reduction by participating entities through these transactions. Existing literature on the carbon emission reduction effects of carbon trading primarily focuses on two dimensions.

Firstly, from an environmental perspective, numerous scholars posit that carbon trading can optimize resource allocation, curb carbon emissions, and improve air quality (Chen&Mu,2023; Dong et al., 2022; Wang et al., 2022; Xu et al., 2020; Fan et al., 2018; Wang&Gao,2018; Martinez et al., 2016) [2–8], thereby facilitating environmentally sustainable development (Cui et al., 2021; Liu et al., 2019) [9,10]. Secondly, on the economic front, a multitude of studies suggest that carbon trading can enhance enterprise investment efficiency (Zhang et al., 2022) [11], promote technological innovation (Qin&Xie, 2023; Liu&Liu, 2023; Zhang&Luo, 2023) [12–14], and boost production levels (Lu, Li, 2023; Hu&Tang, 2022) [15,16]. Consequently, this brings about noteworthy economic benefits (Deng&Zhang, 2019; Yu&Chen, 2017) [17,18], ultimately compelling enterprises to reduce emissions and increase efficiency. Therefore, through the carbon trading market mechanism, businesses can effectively facilitate their energy conservation, emission reduction, and value enhancement.

### 2.2Application of evolutionary game in carbon emission reduction

Evolutionary game theory, premised on the bounded rationality of behavioral agents, examines the dynamic process of individuals within a population achieving a relatively stable state through imitation, learning, and strategic adjustment. Currently, research on corporate carbon emission reduction mostly focuses on incorporating policy makers’ government considerations and the bounded rationality of behavioral agents, thereby distilling the decision-making process of corporate carbon emission reduction into an evolutionary game problem between the government and businesses (Cheng et al., 2023; Guo et al., 2023; Li et al., 2022; Fang et al., 2021) [19–22]. Beyond the government-business dyad, some scholars also engage third-party regulators such as consumers, news media, and the public in the game (Zhao et al., 2023; Meng et al., 2022; Guo et al., 2021; Luo et al., 2020; Tang, 2019; Wang et al., 2019; Xu et al., 2018) [23–29] to explore carbon emission reduction in heavy-polluting industries like coal, cement, and power generation.

### 2.3 Evolutionary game analysis of enterprise carbon emission reduction under carbon trading mechanism

Evolutionary game theory, capable of explaining complex economic phenomena, serves as a vital analytical tool for studying carbon trading markets and is widely applied in coordinating interest conflicts and achieving game equilibria among relevant entities such as central and local governments as well as businesses. In terms of research content, one approach involves constructing evolutionary game models by introducing parameters such as carbon trading prices, government supervision costs, enterprise emission reduction investments, and governance costs to explore the factors influencing corporate carbon emission reduction within the carbon trading mechanism (Yang et al., 2022; Guo et al., 2021; Ryle S. Perera, 2018; Jiao et al., 2017) [30–33]. Another approach focuses on analyzing the impact of government subsidy models, initial carbon emission right allocation methods, and government incentive measures on corporate carbon emission reduction decisions (Ren et al., 2016; Lu et al., 2015; Zhao et al., 2018; Luo, Fan, 2014) [34–37]. In terms of research findings, carbon trading policies can balance profits and business reputation, thereby facilitating effective emission reduction (Wang et al., 2021) [38]. Additionally, carbon trading prices below a critical threshold can stimulate corporate enthusiasm for honest emission reduction (Geng et al., 2022) [39].

The aforementioned literature provides numerous insights for research in this paper, yet there are still some limitations: 1) While most studies focus on carbon emission reduction in heavy-polluting industries such as coal, cement, and power generation, few have directed their attention to Internet companies that have grown to consume substantial amounts of electricity. 2) Despite the introduction of various parameter factors under the carbon trading mechanism, the majority of research only examines the perspective of the government and businesses, neglecting adequate consideration for the carbon market, financial institutions, and the public. Therefore, taking into account constraints from the carbon market, financial institutions, and society, this paper constructs an evolutionary game model for internet companies entering the carbon market. Through numerical simulations, it depicts the evolutionary path of carbon emission reduction, aiming to provide decision-making reference suggestions for the government, internet companies, and other stakeholders to promote sustainable development.

## 3. Game model construction

### 3.1 Problem description and research hypothesis

As the issue of global climate change becomes increasingly urgent, low-carbon economies and sustainable development have become international consensus. As one of the world’s largest emitters of carbon, China has taken proactive measures, setting targets to peak carbon emissions by 2030 and achieve carbon neutrality by 2060. This effort is complemented by the establishment of a national carbon trading market to promote carbon emission reduction. Concurrently, the robust development of the global digital economy and the deepening of the "Digital Silk Road" initiative provide unprecedented opportunities for internet companies. However, with the continuous growth of internet companies in scale and business volume, their carbon emissions also show an upward trend. Although their carbon footprint may be small, their impact on future carbon emission patterns cannot be ignored. To achieve sustainable development, internet companies must assume responsibility for carbon emission reduction and actively participate in carbon trading. Yet, in entering the carbon market, these companies face various constraints from multiple stakeholders, including emission reduction costs, penalties for non-compliance, government subsidies, financing costs, opportunity losses, and reputation losses, which directly influence their decisions on carbon emission reduction and engaging in carbon trading. Therefore, an evolutionary game analysis focused on internet companies entering the carbon market is particularly significant. By deeply analyzing the impact of multiple constraints on companies’ low-carbon emission reduction paths, targeted decision-making references can be provided to internet companies, promoting their sustainable development while demonstrating exemplary and driving roles in low-carbon emission reduction. Ultimately, these efforts will contribute to finding a path that aligns with economic development principles while achieving environmental protection and sustainable development, helping China to achieve its carbon neutrality goals.

#### 3.1.1 Impact of carbon market on carbon emission reduction of internet enterprises

Carbon trading is the abbreviated term for "carbon emission rights trading," originally proposed by American economist John H. Dales in 1968. The Kyoto Protocol, signed in 1997, introduced joint implementation, emissions trading, and clean development mechanisms to address global climate change, further promoting the development of carbon trading. Carbon trading does not involve directly trading greenhouse gases, but rather trading their emission rights, converting greenhouse gases, primarily CO_2_, into CO_2_ equivalents, thus establishing carbon trading. Carbon trading is based on Externality, where addressing greenhouse gases incurs cost differences for businesses, leading to the emergence of carbon trading markets. The fundamental economic principle of the carbon market is to price carbon emission rights through market mechanisms, aiming to minimize the costs of carbon emission reduction. Specifically, the government allocates free carbon quotas to businesses, which then engage in trading based on these allocated quotas. When a company emits less carbon than its quota, it can sell the remainder for revenue. Conversely, if emissions exceed the quota, it must purchase additional quotas to fulfill its environmental responsibilities, effectively compelling companies to reduce carbon emissions. As carbon markets continue to evolve, the proportion of free quotas will gradually decrease, resulting in increasing transaction costs for companies that do not adopt emission reduction measures.

Enterprises participating in carbon trading must comply with the regulatory requirements of the carbon market. First of all, enterprises need to accurately report carbon emission data, which requires them to establish a sound carbon emission data collection and reporting system to ensure the accuracy and integrity of data. Secondly, enterprises need to accept carbon verification by independent third-party institutions, which will verify and review the carbon emission data submitted by enterprises to ensure the authenticity and reliability of the data. Failure to voluntarily conduct carbon verification or to adhere to the standard verification process will result in penalties for non-compliant enterprises, including notifications, sanctions, fines, and inclusion on a blacklist of discredited entities. With the deepening of the construction of the carbon market, the verification, supervision, and punishment of carbon emission management will be further strengthened and strict.

The carbon market presents both challenges and potential opportunities for internet companies. These companies, often with large-scale data centers and servers, face increased carbon emissions due to their surging power consumption. Engaging in carbon trading may incur additional costs, and failure to comply with regulatory requirements can lead to penalties, blacklisting, and damage to corporate reputation and market credibility. However, internet companies possess advantages in the carbon market due to their digitization, networking, and intelligence. Their large-scale data processing and analysis capabilities ensure accurate and complete data reporting, while their innovation in low-carbon technologies not only reduces their carbon footprint but also generates revenue. Given this context, internet companies can strategically choose to enter or not enter the carbon market. Those that do can buy or sell carbon quotas based on demand, receiving free quotas as a base. Those that don’t avoid direct costs but miss out on the economic and environmental benefits. Against this backdrop, the following hypotheses can be proposed:

Hypothesis 1: As limited rational entities, internet companies can strategically choose to enter or not enter the carbon market.
Hypothesis 2: The proportion of free quotas will gradually decrease, leading to increased costs for carbon transactions.
Hypothesis 3: The carbon market will be strictly regulated with significant penalties to ensure fair, transparent, and accurate trading.

#### 3.1.2 Impact of financial institutions on carbon emission reduction of internet enterprises

In order to promote high-quality development of the green and low-carbon economy, financial institutions have increased their credit and product service support for green industries in recent years. As a result, green credit has become a key focus and highlight of financial institution credit allocation. Also known as sustainable financing or environmental financing, green credit refers to banks incorporating environmental monitoring standards, pollution control effectiveness, and ecological environmental protection as important assessment criteria during the loan process(Zhang&Wu,2023) [40]. Consequently, financial institutions are not only focusing on the economic benefits of enterprises but also increasingly emphasizing their performance in environmental protection and sustainable development. To this end, financial institutions are offering financial products such as low-interest and long-term green loans and green bonds to reduce corporate financing costs and encourage long-term carbon emission reduction investments. Additionally, guided by government departments, various documents promoting "pollution reduction, carbon reduction, and synergistic efficiency" have been issued, further encouraging financial institutions to provide more financing support and opportunities for enterprises actively pursuing emission reductions. Therefore, financing difficulties will increase for internet companies that do not prioritize carbon emission reductions, leading to the formulation of Hypothesis 4.

Hypothesis 4: Financial institutions provide more financing incentives and support for internet companies that prioritize environmental protection and sustainable development.

#### 3.1.3 Impact of the public on carbon emission reduction of internet enterprises

As global climate change issues become increasingly urgent, the environmental awareness of the general public continues to grow, forming a significant driving force for carbon emission reduction in internet companies. Firstly, from the perspective of consumer choices, the public is paying closer attention to corporate environmental responsibility when selecting products and services. They are more willing to choose and pay higher prices for products and services offered by companies committed to low-carbon emission reduction and sustainable development(Zhou,2022) [41]. Therefore, companies that proactively assume environmental responsibilities enjoy a higher reputation, gaining consumer trust and attracting more customers. Secondly, from the perspective of public opinion, internet companies that perform poorly in carbon emission reduction may face criticism from the public, potentially damaging their reputation and brand value. Conversely, those who actively adopt carbon emission reduction measures cultivate a positive corporate image and strengthen their brand’s market competitiveness. Consequently, internet companies that proactively assume environmental responsibilities gain an advantage in public opinion, thereby enhancing their brand image and market competitiveness. Therefore, Hypothesis 5 is proposed.

Hypothesis 5: The public demands a high level of environmental responsibility from internet companies.

**Fig 1:**
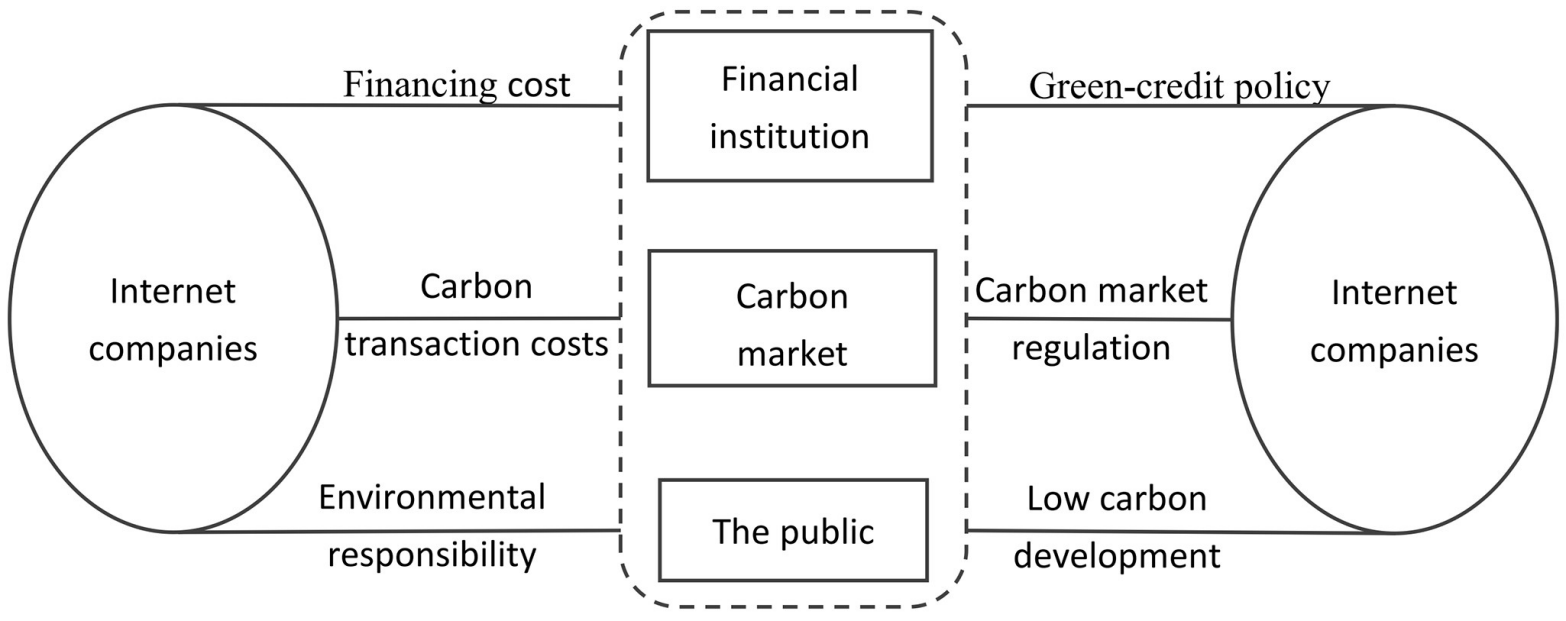
Model mechanism.

The carbon market effectively drives enterprises to reduce carbon emissions through market mechanisms of total quantity control and quota trading. However, as the carbon market becomes increasingly sophisticated and the allocation of free quotas gradually decreases, the cost of carbon transactions continues to rise, and carbon market supervision becomes increasingly stringent, with penalty intensity consistently escalating. Meanwhile, guided by the government, financial institutions are placing greater emphasis on the green credibility of enterprises, further exacerbating the challenges in corporate financing. Coupled with the growing public attention to corporate environmental responsibility, it has become imperative for internet companies to reduce pollution and decrease carbon emissions. In summary, under the multiple constraints of the carbon market, financial institutions, and the public, constructing an evolutionary game model for carbon emission reduction in internet companies can facilitate sustainable corporate development. The model mechanism is illustrated in Fig 1.

### 3.2 Parameter definition and return matrix

Based on the aforementioned model mechanism and research hypotheses, we establish the following parameters to describe the game situation between internet companies A and B in the carbon market, assuming that all parameters are positive constants. In the game, both internet companies A and B have two strategies: to enter the carbon market or not to enter the carbon market. Let the probability of company A entering the carbon market be x, and the probability of not entering be 1-x. Similarly, let the probability of company B entering the carbon market be y, and the probability of not entering be 1-y. These probabilities are functions of time t, reflecting the strategic choices of enterprises at different time points.

When a company decides to enter the carbon market, it incurs certain costs, including expenses for monitoring and reporting carbon emission data, carbon quota transaction costs, and expenses for technological transformation and energy transition. To simplify the model, we represent the total of these costs as C. To encourage enterprises to reduce carbon emissions, the government implements measures such as free carbon quota allocation, carbon emission reduction incentive mechanisms, green fund and project support, tax incentives, and exemptions to promote low-carbon development. We assume that the subsidy provided by the government to enterprises actively participating in carbon trading is represented by S. Additionally, the carbon market imposes penalties on non-compliant transactions, with fines represented by I. Companies that choose not to enter the carbon market may face various opportunity losses, such as missing out on carbon market profits, financing and investment opportunities, and brand value enhancement. Therefore, we represent the opportunity losses incurred by non-participating companies due to participating companies entering the carbon market as L, the increment in investment and financing costs as M, and the loss of reputation gained by not entering the carbon market as D.

In addition, when a company chooses to enter the carbon market, its actions will trigger changes in market share and carbon quota allocation, resulting in differences in the benefits obtained by each enterprise. Therefore, we assume that when Internet company B enters or does not enter the carbon market, the benefits obtained by Internet company A are represented by G1 and G2, respectively (where G1 < G2). Similarly, when Internet company A enters or does not enter the carbon market, the benefits obtained by Internet company B are represented by U1 and U2, respectively (where U1 < U2). The parameter settings are shown in Table 1.

**Table 1:** Parameter symbol definition.

parameter	meaning
G_1_	B into the carbon market, A of earnings
G_2_	B does not enter the carbon market, A of earnings
U_1_	A into the carbon market, B of the earnings
U_2_	A does not enter the carbon market, B of the earnings
C	The total cost of entering the carbon market
S	Government subsidies for actively reducing emissions for enterprises
I	Carbon market fines for non-compliant transactions
L	Entering the carbon market loses opportunities to companies that do not enter
M	Incremental financing costs brought to non entering enterprises by entering the carbon market
D	Loss of goodwill gained from not entering the carbon market

Based on externality and sustainable development theories, an evolutionary game model is constructed to explore the decision-making process of internet companies entering the carbon market, considering constraints from the carbon market, financial institutions, and the public. Both Internet companies A and B have two strategic choices: "entering" or "not entering" the carbon market. The utilities of these two strategic choices for both Internet companies A and B are calculated separately, forming a payoff matrix as shown in Table 2.

**Table 2:** Evolutionary game payoff matrix.

Game player		B	
		Enter,y	No enter,1-y
A	Enter,x	G_1_+S-C-I,U_1_+S-C-I	G_2_+S-C-I,U_1_-M-L-D
	No enter,1-x	G_1_-M-L-D,U_2_+S-C-I	G_2_-D,U_2_-D

## 4. Game model analysis

### 4.1 Equilibrium point of the evolutionary game system

In the evolutionary game system composed of internet companies A and B, the initial strategy distribution and proportions are as follows: the probability of A entering the carbon market is x, and the probability of not entering is 1-x; the probability of B entering the carbon market is y, and the probability of not entering is 1-y. Over time, A and B will adjust their strategies based on their own and others’ payoffs. If they find that choosing to "enter" the carbon market yields greater benefits, they will imitate this strategy. This process of strategy adjustment can be precisely described through replicator dynamics equations, which are mathematical models that depict how strategy proportions change over time. In the process of evolution, A and B will continuously adjust their strategies according to the replicator dynamics equations to achieve higher expected payoffs. Ultimately, their strategic choices will reach an equilibrium state, which is the equilibrium point of the evolutionary game system. Therefore, the equilibrium point of the evolutionary game system can be determined by solving the replicator dynamics equations.

1)Replication Dynamics Equation for Internet Company A’s Game Model

Let the expected payoffs for Internet Company A when adopting the strategies of "entering" and "not entering" the carbon market be represented by E_a1_ and E_a2_, respectively, and let the average expected payoff be represented by E_a_. Then we have:

$$E_{a1}=y\left(G_{1}+S-C-I\right)+\left(1-y\right)\left(G_{2}+S-C-I\right)$$ (1)

$$E_{a2}=y\left(G_{1}-M-L-D\right)+\left(1-y\right)\left(G_{2}-D\right)$$ (2)

$$E_{a}=xE_{a1}+\left(1-x\right)E_{a2}$$ (3)

The replication dynamics of Internet Company A’s strategic choices can be expressed as:

$$F\left(x\right)=\frac{dx}{dt}=x\left(E_{a1}-E_{a}\right)=x\left(1-x\right)\left[yL+yM+S+D-C-I\right]$$ (4)

2)Replication Dynamics Equation for Internet Company B’s Game Model

Let the expected payoffs for Internet Company B when adopting the strategies of "entering" and "not entering" the carbon market be represented by E_b1_ and E_b2_, respectively, and let the average expected payoff be represented by E_b_. Then we have:

$$E_{b1}=x\left(U_{1}+S-C-I\right)+\left(1-x\right)\left(U_{2}+S-C-I\right)$$ (5)

$$E_{b2}=x\left(U_{1}-M-L-D\right)+\left(1-x\right)\left(U_{2}-D\right)$$ (6)

$$E_{b}=yE_{b1}+\left(1-y\right)E_{b2}$$ (7)

The replication dynamics of Internet Company B’s strategic choices can be expressed as:

$$F\left(y\right)=\frac{dy}{dt}=y\left(E_{b1}-E_{b}\right)=y\left(1-y\right)\left[xL+xM+S+D-C-I\right]$$ (8)

3)Equilibrium Points of Evolutionary Game System

Equilibrium points in an evolutionary game system signify a state where the strategic choices of all participants have reached a balance. At these equilibrium points, the proportions of various strategies no longer change over time, i.e., dx/dt = 0 and dy/dt = 0. In the evolutionary game system composed of internet companies A and B, the condition for achieving an equilibrium point is that the replicator dynamics Eq (4) equals 0 and Eq (8) equals 0. By solving these two equations simultaneously, we obtain Eq (9).


$$\left\{\begin{array}{c} F\left(x\right)=\frac{dx}{dt}=x\left(1-x\right)\left[yL+yM+S+D-C-I\right]=0\\ F\left(y\right)=\frac{dy}{dt}=y\left(1-y\right)\left[xL+xM+S+D-C-I\right]=0 \end{array}\right.$$ (9)

By solving Eq (9), the equilibrium points of the system are obtained as: (0,0), (0,1), (1,0), (1,1), and (x*, y*), where the expressions for x* and y* are given by Eq (10).


$$\left\{\begin{array}{c} x^{*}=\frac{C+I-S-D}{L+M}\\ y^{*}=\frac{C+I-S-D}{L+M} \end{array}\right.$$ (10)

### 4.2 Stability analysis of the equilibrium points

The equilibrium points of an evolutionary game system do not necessarily correspond to Evolutionarily Stable Strategies (ESS). Equilibrium points merely indicate a balanced state of strategy distribution but do not guarantee stability. That is, equilibrium points may not be resilient to small mutations or perturbations. In contrast, Evolutionarily Stable Strategies possess greater stability and resistance to interference. They not only yield higher payoffs in the current environment but also maintain their dominant position in the face of invasions from other mutant strategies, thereby promoting the stable evolution of the system. Therefore, it is crucial to analyze the stability of the system’s equilibrium points.

According to Lyapunov stability theory, for continuous-time linear dynamic systems, if the determinant of the system’s Jacobian matrix at an equilibrium point is positive (det T > 0) and the trace of the matrix is negative (tr T < 0), then that equilibrium point is locally asymptotically stable, which corresponds to an Evolutionarily Stable Strategy (ESS). Specifically, a positive determinant (det T) indicates that the system’s state at the equilibrium point is elliptical, meaning that the system’s state oscillates periodically in the vicinity of that point. A negative trace (tr T) indicates that the oscillation of the system’s state at the equilibrium point is decaying, meaning that over time, the system’s state tends towards that equilibrium point. These two conditions together guarantee the local stability and convergence of the equilibrium point, thus qualifying it as an Evolutionarily Stable Strategy (ESS).

1)Calculate the partial derivatives for the replication dynamic equations F(x) and F(y):

$$\left\{\begin{array}{c} \frac{\partial F\left(x\right)}{\partial x}=\frac{\partial x\left(1-x\right)\left[yL+yM+S+D-C-I\right]}{\partial x}\\ \frac{\partial F\left(x\right)}{\partial y}=\frac{\partial x\left(1-x\right)\left[yL+yM+S+D-C-I\right]}{\partial y}\\ \frac{\partial F\left(y\right)}{\partial x}=\frac{\partial y\left(1-y\right)\left[xL+xM+S+D-C-I\right]}{\partial x}\\ \frac{\partial F\left(y\right)}{\partial y}=\frac{\partial y\left(1-y\right)\left[xL+xM+S+D-C-I\right]}{\partial y} \end{array}\right.$$ (11)

2)Build the Jacobian matrix T using Eq (11):

$$T=\left[\begin{array}{cc} \frac{\partial F\left(x\right)}{\partial x} & \frac{\partial F\left(x\right)}{\partial y}\\ \frac{\partial F\left(y\right)}{\partial x} & \frac{\partial F\left(y\right)}{\partial y} \end{array}\right]=\left[\begin{array}{cc} C_{11} & C_{12}\\ C_{21} & C_{22} \end{array}\right]$$ (12)

3)Solve Eq (11) and substitute the obtained results into Eq (12) to obtain the expressions for each element of the Jacobian matrix T.


$$\left\{\begin{array}{c} C_{11}=\left(1-2x\right)\left[yL+yM+S+D-C-I\right]\\ C_{12}=\text{x}\left(1-x\right)\left[L+M\right]\\ C_{21}=\text{y}\left(1-y\right)\left[L+M\right]\\ C_{22}=\left(1-2y\right)\left[xL+xM+S+D-C-I\right] \end{array}\right.$$ (13)

4)The determinant of the matrix is:

$$det\;T=\left[\begin{array}{cc} C_{11} & C_{12}\\ C_{21} & C_{22} \end{array}\right]=C_{11}C_{22}-C_{12}C_{21}$$ (14)

5)The trace of the matrix is:

$$tr\;T=C_{11}{+C}_{22}$$ (15)

6)By substituting the equilibrium points (0,0), (0,1), (1,0), (1,1), and (x*, y*) of the evolutionary game system into Eq (13), we can obtain the values of each element of the corresponding Jacobian matrix at these equilibrium points. Then, by substituting these values into Eqs (14) and (15), we can calculate the determinant and trace of the Jacobian matrix. The specific results are shown in Table 3.

**Table 3:** Equilibrium point values.

	C_11_	C_12_	C_21_	C_22_	det T	trT
(0,0)	S+D-C-I	0	0	S+D-C-I	(S+D-C-I)^2^	2(S+D-C-I)
(0,1)	L+M+S+D-C-I	0	0	C+I-S-D	(C+I-S-D)(L+M+S+D-C-I)	L+M
(1,0)	C+I-S-D	0	0	M+D+L+S-C-I	(C+I-S-D)(L+M+S+D-C-I)	L+M
(1,1)	C+I-S-M-D-L	0	0	C+I-M-L-D-S	(C+I-S-M-D-L)^2^	2(C+I-S-M-D-L)
(x*,y*)	0	(C+I-S-D)(L+M+S+D-C-I)/L+M	(C+I-S-D)(L+M+S+D-C-I)/L+M	0	-[(C+I-S-D)(L+M+S+D-C-I)/L+M]^2^	0

According to the results in Table 3, only (0,0) and (1,1) are equilibrium points that correspond to the Evolutionarily Stable Strategies (ESS) of the system. The reasoning is as follows: when the local equilibrium points are (0,1) and (1,0), their trace trT = L + M > 0 because in the parameter settings, L and M are assumed to be positive. When the local equilibrium point is (x*, y*),trT = 0. These two scenarios clearly do not satisfy the conditions for Evolutionarily Stable Strategies, which require det T> 0 and trT < 0. Therefore, the equilibrium points (0,1), (1,0), and (x*, y*) are not Evolutionarily Stable Strategies equilibrium points of the system.

### 4.3 Analysis of the evolutionary game results

When the equilibrium point is (0,0), the strategy choice combination for Internet companies A and B is (not to enter, not to enter) the carbon market. At this time, the conditions for the system’s evolutionary stability are (S + D − C − I)^2^ > 0 and 2(S + D − C − I) < 0, which simplifies to S + D − C − I < 0. This means that when the sum of government subsidies (S) for entering the carbon market and the loss of reputation (D) for not entering the carbon market is less than the total cost (C) of entering the carbon market and the penalty (I) for non-compliance, both Internet companies A and B choose not to enter the carbon market. Given that all the parameters are assumed to be constants greater than 0, further interpretation of this condition reveals two insights: Firstly, S < C + I, which indicates that if the government subsidy for entering the carbon market is less than the sum of the total cost and penalty for entering, Internet companies choose not to enter the carbon market; secondly, D < C + I, which suggests that if the loss of reputation for not entering the carbon market is less than the sum of the total cost and penalty for entering, companies prefer to maintain the status quo and not participate in carbon trading.

It can be inferred that economic interests and reputation losses are crucial factors in the strategic decision-making process for Internet companies A and B when determining whether to enter the carbon market. If government subsidies are insufficient to offset the costs and risks associated with entering the carbon market, companies lack sufficient motivation to participate in carbon trading. Similarly, if the reputation loss incurred by maintaining the status quo is less than the total cost and penalty of entering the carbon market, companies are more inclined to maintain their current situation. This reflects the need for businesses to strike a balance between economic benefits and social reputation when making decisions. If the government can develop more attractive subsidy policies, and the public can further enhance their attention to the emission reduction effects of participating in carbon trading, it may incentivize more companies to engage in carbon trading and thereby promote the development of a low-carbon economy.

When the equilibrium point is (1,1), the strategy choice combination for Internet companies A and B is (enter, enter) the carbon market. At this time, the conditions for the system’s evolutionary stability are (C + I − S − L − D − M)^2^ > 0 and 2(C + I − S − L − D − M) < 0, which simplifies to C + I − S − L − D − M < 0. After rearranging, we obtain C + I < S + L + D + M. This indicates that when the sum of the total cost (C) of entering the carbon market and the penalty (I) for non-compliance is less than the sum of government subsidies (S) for entering the carbon market, the incremental financing costs (M), opportunity losses (L), and reputation losses (D) incurred by not entering the carbon market, both companies will adopt a strategy of entering the carbon market. This suggests that companies are willing to participate in carbon trading when the benefits of government subsidies, avoided financing costs, opportunity gains, and reputation gains outweigh the costs and risks associated with entering the carbon market.

From the decision-making process of businesses, it is evident that economic interests and risk assessment are crucial considerations when determining whether to engage in low-carbon emission reduction activities. Companies are motivated to actively participate in carbon trading when they assess that the economic benefits of entering the carbon market outweigh the associated risks and costs. This decision is not solely based on immediate financial gains, but rather reflects a strategic forecast and judgment of the future trend of a low-carbon economy. For companies that firmly believe that a low-carbon economy will become the mainstream development trend in the future, participating in carbon trading can be a critical aspect of maintaining a competitive edge in the fierce market competition. Especially when the sum of the total cost of entering the carbon market and potential non-compliance penalties is less than the combination of government subsidies, incremental financing costs, opportunity losses, and reputation losses incurred by not entering the carbon market, companies are more compelled to actively engage in carbon trading. Therefore, in such scenarios, businesses recognize that participating in carbon trading not only yields greater economic benefits but also effectively manages various risks associated with carbon emissions, further reinforcing their profound understanding of the future trend of a low-carbon economy. To encourage more companies to embrace low-carbon practices, governments and relevant institutions must actively contribute by developing more attractive support policies, assisting businesses in reducing financing costs, and fostering a favorable environment that stimulates corporate enthusiasm for proactive emission reductions and sustainable economic development.

## 5. Numerical simulation analysis

### 5.1 Data source and parameter settings

To gain a deeper understanding of the evolutionary relationships between the strategic choices of game players, as well as the impacts of various factor changes under multiple constraints such as emission reduction costs, penalties for violations, government subsidies, financing costs, opportunity losses, and reputation losses, this paper employs Python 3.8.2 software to conduct numerical simulation modeling and visualize the dynamic behaviors of game players.

According to data disclosed by the Shanghai Environment and Energy Exchange, the daily closing price of carbon emission quotas in the national carbon market fluctuated slightly between 55 yuan/ton and 62 yuan/ton in 2022, with an average annual transaction price of 55.30 yuan/ton. Therefore, the emission reduction cost (C) was set at 55 yuan/ton CO_2_. Based on 30 sets of data with penalty records disclosed by the Ministry of Ecology and Environment, after converting to the average penalty amount, the fine for carbon trading violations was 0.105 yuan/ton CO_2_, thus setting the penalty for violations (I) at 0.1 yuan/ton CO_2_. According to the "2022 Implementation Guidelines for the Development Fund of Beijing’s High-Tech and High-End Industries", a 30% subsidy of total investment is provided for emission reduction incentives, so the government subsidy (S) was set at 16 yuan/ton CO_2_. Shen et al. (2012) argued that if enterprises do not reduce emissions, negative public evaluation can result in losses amounting to 23.89% of their accumulated excess earnings [42]. Therefore, the opportunity loss (L) for not entering the carbon market was set at 24 yuan/ton CO_2_. Zhou (2022) believed that consumers are more willing to purchase products from low-carbon enterprises [41], thus setting the reputation loss (D) for not entering the carbon market at 10 yuan/ton CO_2_. In 2021, the People’s Bank of China launched carbon emission reduction support tools, which encouraged low-carbon enterprises to obtain loan interest rates that are 0.73% lower than those for ordinary enterprise loans. Therefore, the increment in financing costs (M) was set at 0.4 yuan/ton CO_2_. Drawing on the research views of Qin and Li (2018), the net benefits of carbon emission reduction measures are nearly equal to the cost of carbon reduction [43], so the benefits obtained by enterprises participating in carbon trading (G_2_, U_2_) were set at 120 yuan/ton CO_2_. Levinson (2019) believed that the comprehensive benefits of emission reduction measures adopted by enterprises are about 20% higher than those in non-emission reduction scenarios [44]. Therefore, the benefits obtained by enterprises not participating in carbon trading (G_1_, U_1_) were set at 100 yuan/ton CO_2_. Under initial conditions, enterprises have an equal probability of entering or not entering the carbon market, so x = y = 0.5. The specific parameter values are shown in Table 4.

**Table 4:** Setting of parameter values.

	parameter	value
parameter values	emission reduction costs(C)	55yuan/tonCO_2_
	penalty for non-compliance(I)	0.1yuan/tonCO_2_
	government subsidies(S)	16yuan/tonCO_2_
	opportunity losses(L)	24yuan/tonCO_2_
	goodwill losses(D)	10yuan/tonCO_2_
	incremental financing costs(M)	0.4yuan/tonCO_2_
	Participate in trading earnings(G_2_, U_2_)	120yuan/tonCO_2_
	Do not participate in the transaction proceeds(G_1_, U_1_)	100yuan/tonCO_2_
initial condition	Probability of companies choosing to enter the carbon market(x, y): 0.5 Probability of not entering the carbon market (1-x, 1-y): 0.5	
simulation software	Python 3.8.2	

Note: See "Data Sources" in the last part of this manuscript for data sources.

### 5.2 Impact of initial willingness on stable strategies

**Fig 2:**
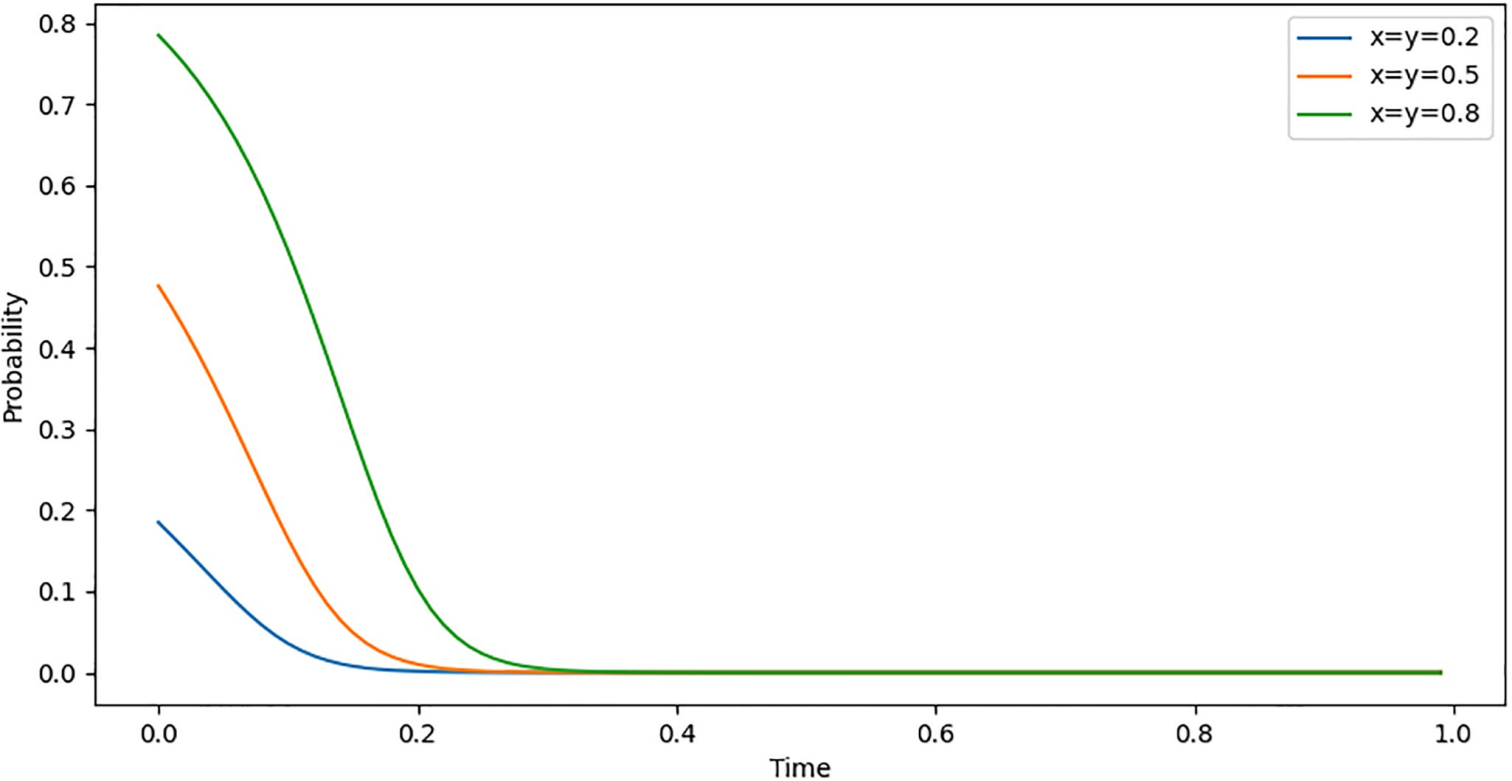
Evolutionary path of initial willingness change.

The initial willingness of Internet companies to enter the carbon market is divided into three levels: "high, medium, and low", with corresponding probabilities of 0.8, 0.5, and 0.2. By combining the set parameters, the evolutionary path of Internet companies entering the carbon market under three different initial willingness levels can be depicted. The vertical axis represents the probability of Internet companies entering the carbon market, while the horizontal axis represents time, as shown in Fig 2.

By observing Fig 2, we can see that under the three initial willingness settings of "high, medium, and low", the system’s evolutionary paths quickly converge to the state of (0,0) within a very short period of time. This phenomenon reveals an important observation, namely that the carbon emission reduction behavior of Internet companies exhibits significant externality. Here, the term "Externality" refers to a clear lack of motivation for Internet companies to participate in carbon trading and actively pursue carbon emission reduction in the absence of explicit constraints or incentive mechanisms. Typically, the costs of carbon emission reduction must be borne by the companies themselves, while the environmental benefits resulting from emission reduction are shared by the entire society. This asymmetry between costs and benefits leads to a lack of motivation for companies to actively engage in carbon emission reduction without sufficient external incentives. Therefore, despite potential differences in initial willingness, the probability of Internet companies participating in the carbon market will rapidly decrease to 0 in the absence of effective constraints and incentive mechanisms.

It can be inferred from this that relying solely on corporate social responsibility or moral constraints may not effectively encourage Internet companies to participate in carbon emission reduction. Therefore, under the multiple constraints of the carbon market, financial institutions, and the general public, it is crucial to adjust established parameters and seek internal drivers that can promote carbon emission reduction among Internet companies.

### 5.3 Impact of multiple constraints on stable strategies

#### 5.3.1 Impact of emission reduction costs on evolutionary paths

**Fig 3:**
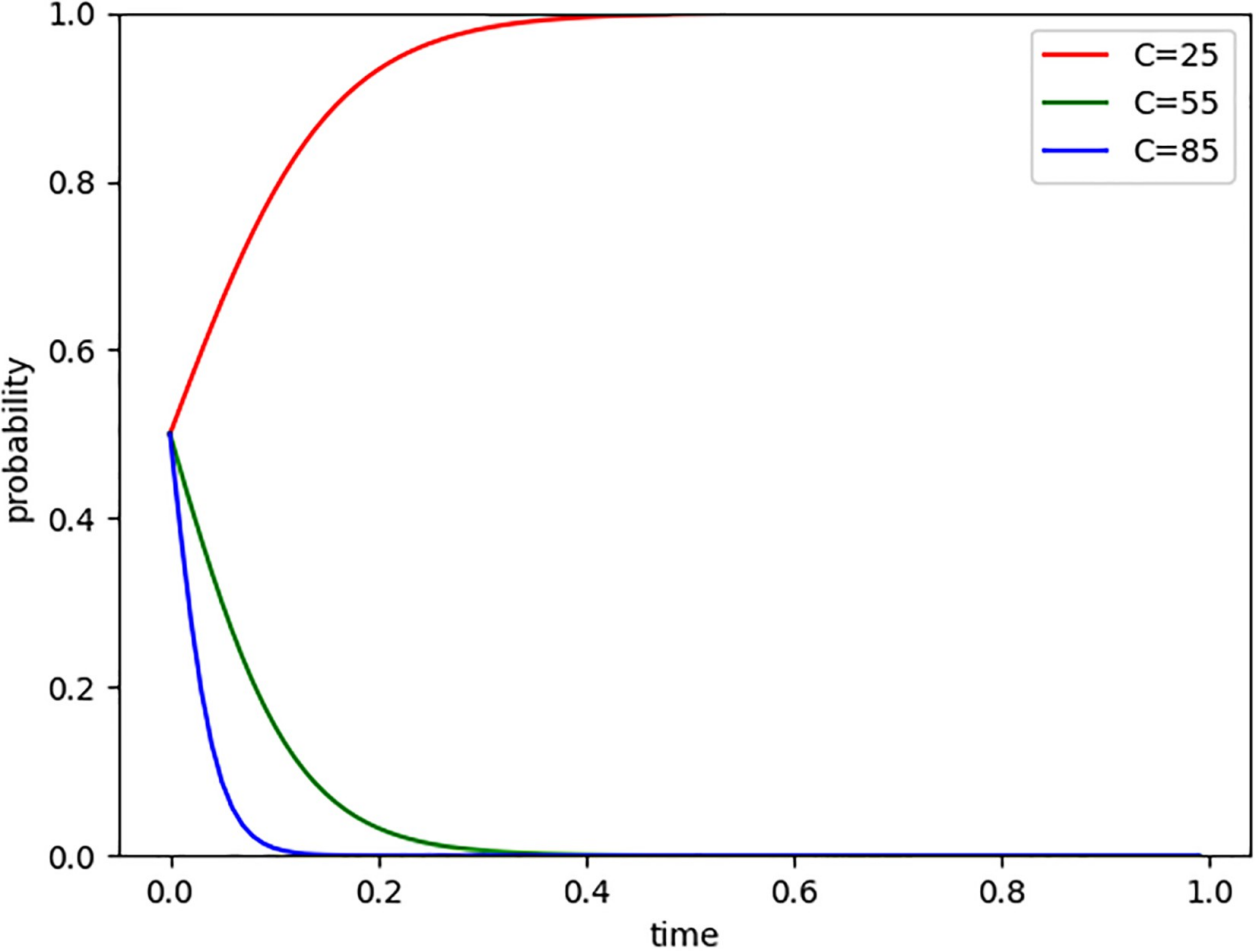
Evolutionary paths of emission reduction cost changes.

As the global climate change issue becomes increasingly urgent, the carbon market, as an important tool to address this challenge, is gradually maturing. At the same time, the goal of "carbon neutrality" is also approaching, which has led to a decreasing allocation of free carbon quotas and subsequently increased the cost of emission reduction for companies participating in carbon trading. To better understand how changes in emission reduction costs affect the attitudes and strategies of Internet companies entering the carbon market, we observed the impact by setting emission reduction costs (C) at three different levels: 25, 55, and 85. As shown in Fig 3, when C = 25, Internet companies quickly converge towards (1,1), indicating their willingness to enter the carbon market when emission reduction costs are low. However, when C = 55 and C = 85, Internet companies quickly converge towards (0,0), indicating their reluctance to enter the carbon market as emission reduction costs increase. Moreover, the higher the emission reduction cost, the shorter the evolution time, and the stronger the companies’ reluctance to enter the carbon market.

This finding aligns with the general principles of market development and confirms that the cost of emission reduction is a crucial consideration for businesses engaging in carbon trading. However, this does not suggest that the carbon trading mechanism lacks incentives for companies. On the contrary, as the carbon market becomes increasingly sophisticated, the gradual reduction of free carbon quotas compels businesses to shoulder higher costs for emission reduction. Consequently, companies are compelled to adopt environmentally friendly production strategies and technologies, enhance energy efficiency, and reduce carbon emissions to meet this challenge. From another perspective, once a company successfully reduces its carbon footprint, it can generate economic returns by selling its surplus carbon quotas to other companies in need. In essence, the carbon trading market mechanism offers businesses a novel profit model that enables them to balance social responsibility with economic gains. While the increased cost of emission reduction may pose short-term pressure on businesses, it ultimately serves as a significant driver for promoting sustainable development among internet companies in the long run.

#### 5.3.2 Impact of penalty for non-compliance on evolutionary paths

**Fig 4:**
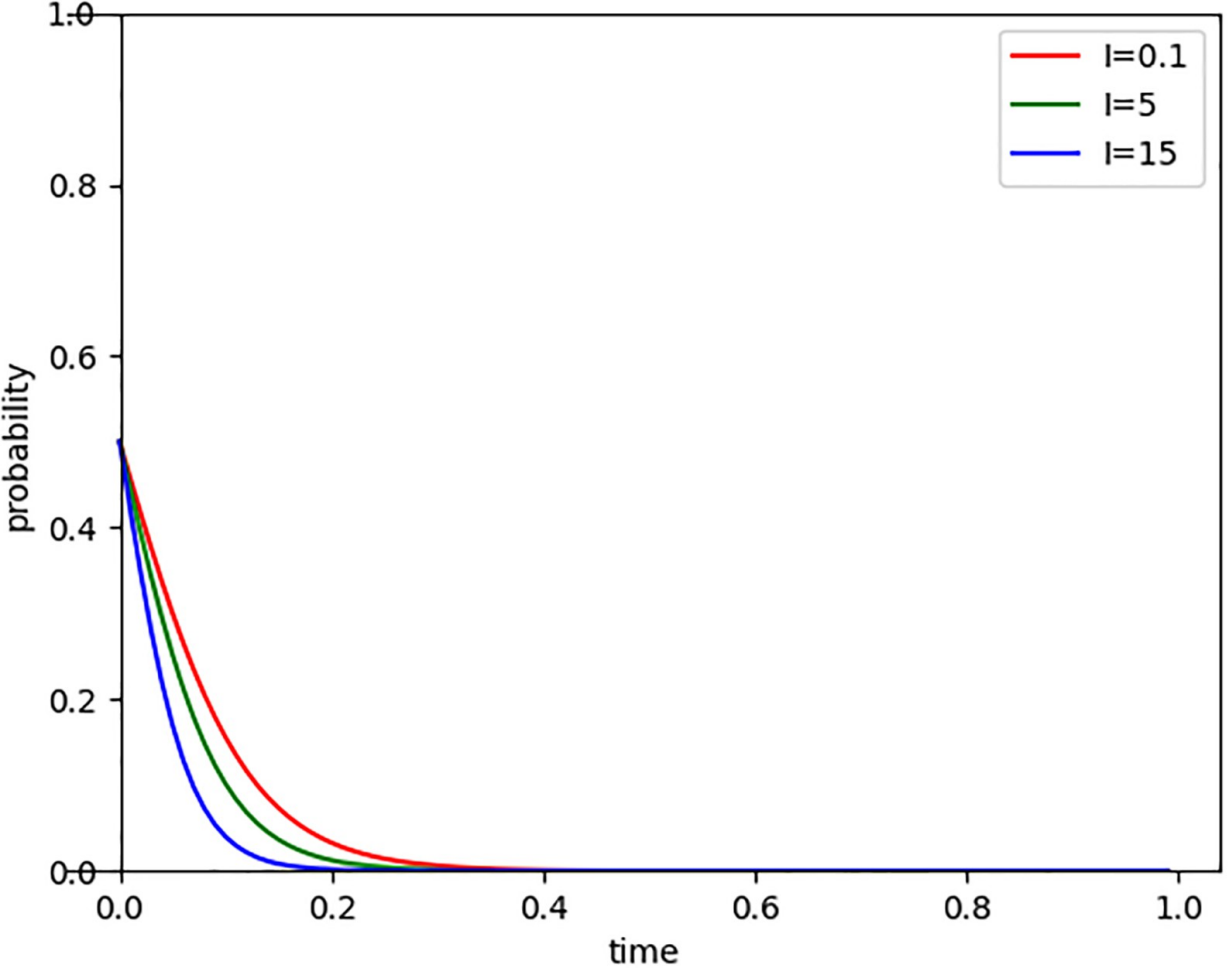
Evolutionary paths of penalty for non-compliance changes.

With the continuous development and improvement of the carbon market, regulatory bodies may increase the penalty for non-compliance in carbon trading, leading to a corresponding increase in fine amounts. By setting the penalty for non-compliance (I) at 0.1, 5, and 15 respectively, we observed the evolutionary path of Internet companies entering the carbon market, as shown in Fig 4. The results indicate that the higher the penalty for non-compliance in the carbon market, the shorter the time for system evolution, and the less willing Internet companies are to enter the carbon market. Because high fines not only increase the economic pressure on companies but also make them more cautious in deciding whether to participate in carbon trading. It reflects a trade-off: while strict penalties can push companies to reduce carbon emissions, excessively high fines may also suppress their enthusiasm. Therefore, when formulating carbon market policies, regulatory bodies need to carefully consider the setting of fine amounts to ensure that they can effectively promote corporate participation without excessively dampening their enthusiasm.

#### 5.3.3 Impact of government subsidies on evolutionary paths

**Fig 5:**
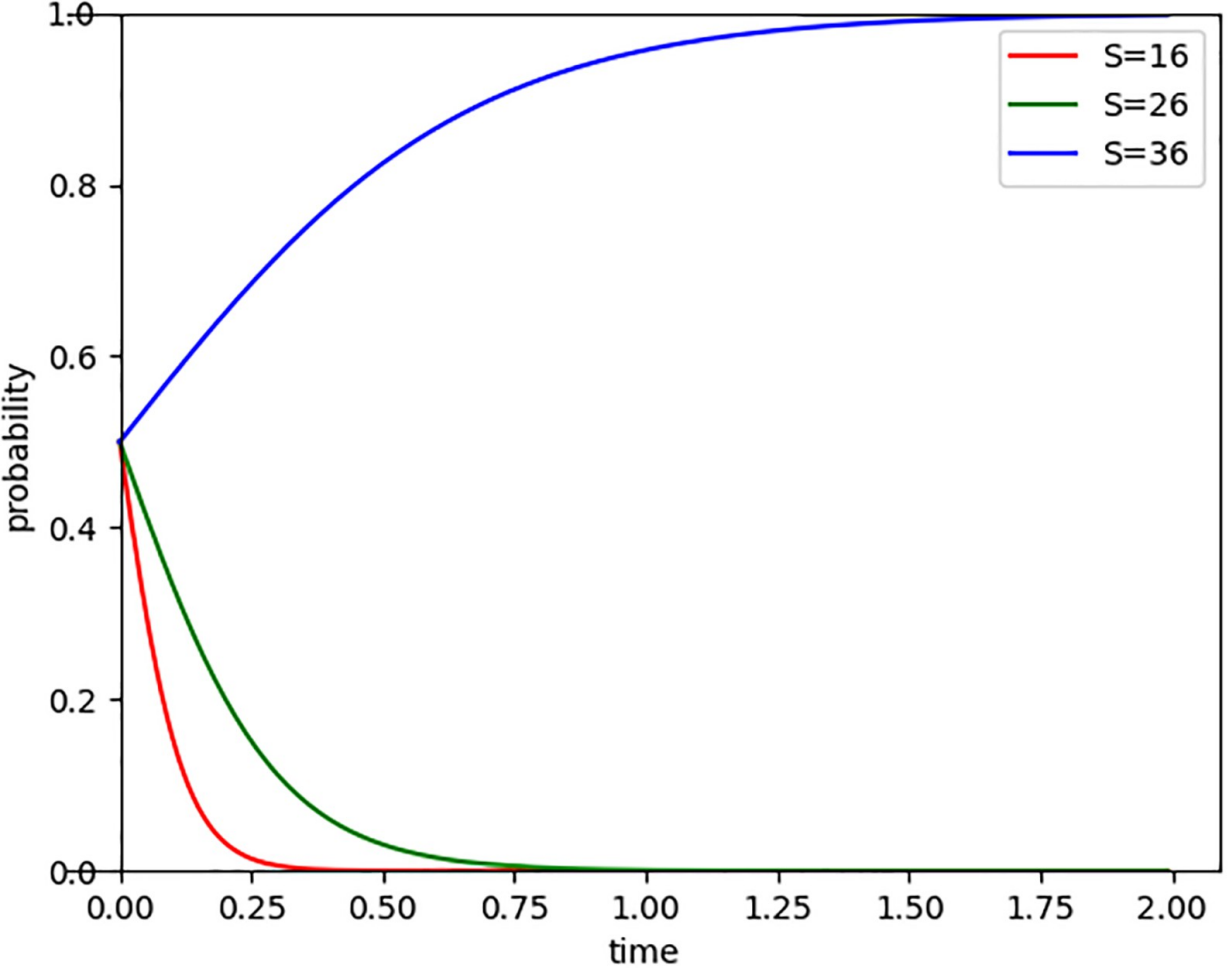
Evolutionary paths of government subsidy changes.

With the deepening of the concepts of green, low-carbon, and sustainable development, the government’s support for low-carbon enterprises has been increasing, leading to a corresponding increase in the scale and intensity of government subsidies. By setting the government subsidy (S) at 16, 26, and 36 respectively, we observed the evolutionary path of Internet companies entering the carbon market, as shown in Fig 5. The results indicate that the more subsidies the government provides, the longer the time for Internet companies to evolve and enter the carbon market. Especially when the government subsidy reaches 36, Internet companies gradually tend to actively participate in carbon trading. However, when the subsidy amount is 16 and 26, although the increase in government subsidies prolongs the time for companies to participate in carbon trading, ultimately the limited subsidies may still result in the loss of motivation for carbon emission reduction among enterprises. Therefore, while government subsidies can incentivize corporate participation in carbon trading to some extent, excessively high subsidies may also create dependency among enterprises. Once the subsidies are reduced or eliminated, their motivation for emission reduction may also weaken.

#### 5.3.4 Impact of financing costs on evolutionary paths

**Fig 6:**
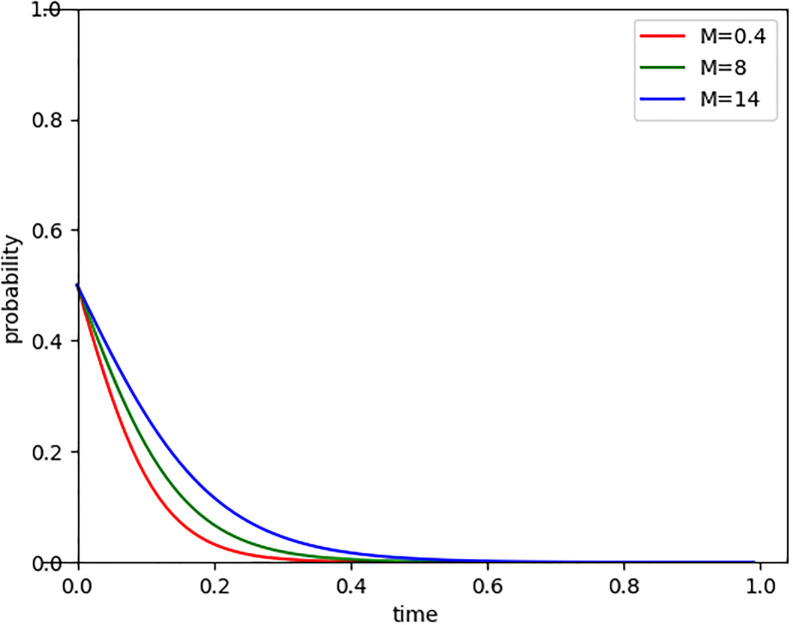
Evolutionary paths of financing cost increment changes.

In November 2021, the People’s Bank of China launched carbon emission reduction support tools, which enabled low-carbon enterprises to obtain loan interest rates that were approximately 0.73% lower than those for ordinary enterprises. From this, we can infer that low-carbon enterprises have lower financing costs. By setting the increment of financing cost (M) at 0.4, 8, and 14 respectively, we observed the evolutionary path of Internet companies entering the carbon market, as shown in Fig 6. The results indicate that the higher the financing cost, the longer the time for Internet companies to evolve and participate in carbon trading, and the stronger their motivation for carbon emission reduction. When faced with high financing costs, companies often seek to reduce operating costs and risks, and adopt measures such as reducing energy consumption, improving production processes, and using clean energy to reduce carbon emissions. By doing so, they can not only obtain financing preferences but also achieve low-carbon transformation. Therefore, high financing costs can serve as one of the catalysts forcing Internet companies to reduce carbon emissions.

#### 5.3.5 Impact of opportunity loss on evolutionary paths

**Fig 7:**
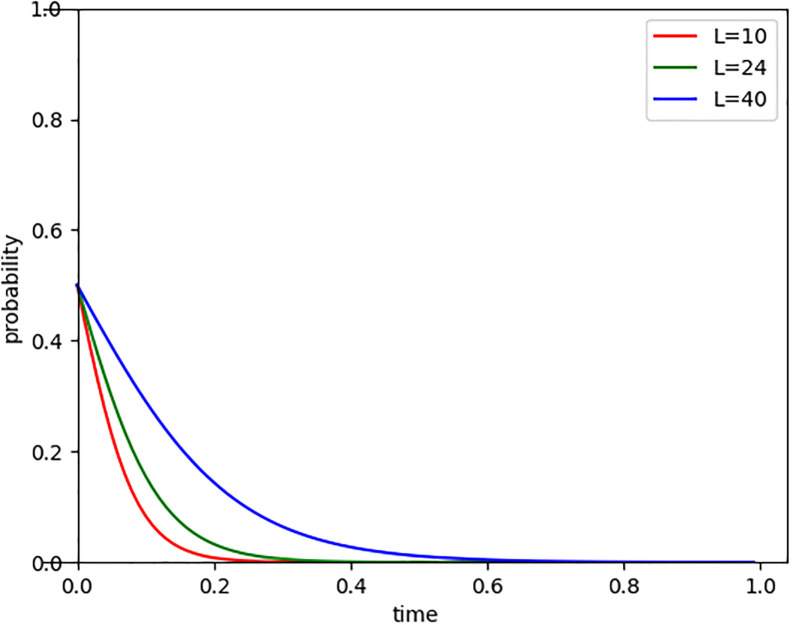
Evolutionary paths of opportunity loss changes.

Companies that do not enter the carbon market, may face various opportunity losses, such as missing out on carbon market profits, financing and investment opportunities, and brand value enhancement. Setting opportunity losses (L) at 4, 24, and 44, we observed the evolution path of Internet companies entering the carbon market, as shown in Fig 7. The results indicate that the greater the opportunity loss, the stronger the determination and duration of Internet companies to participate in carbon trading, demonstrating a stronger willingness to reduce carbon emissions. It suggests that companies that choose to avoid the carbon market may miss out on opportunities beneficial to their own development. As the role of the carbon market becomes increasingly prominent in the economic sphere, companies that do not participate may risk becoming marginalized. Therefore, companies must carefully consider the significance of participating in the carbon market and be aware of the potential long-term losses associated with staying out.

#### 5.3.6 The impact of goodwill loss on evolutionary paths

**Fig 8:**
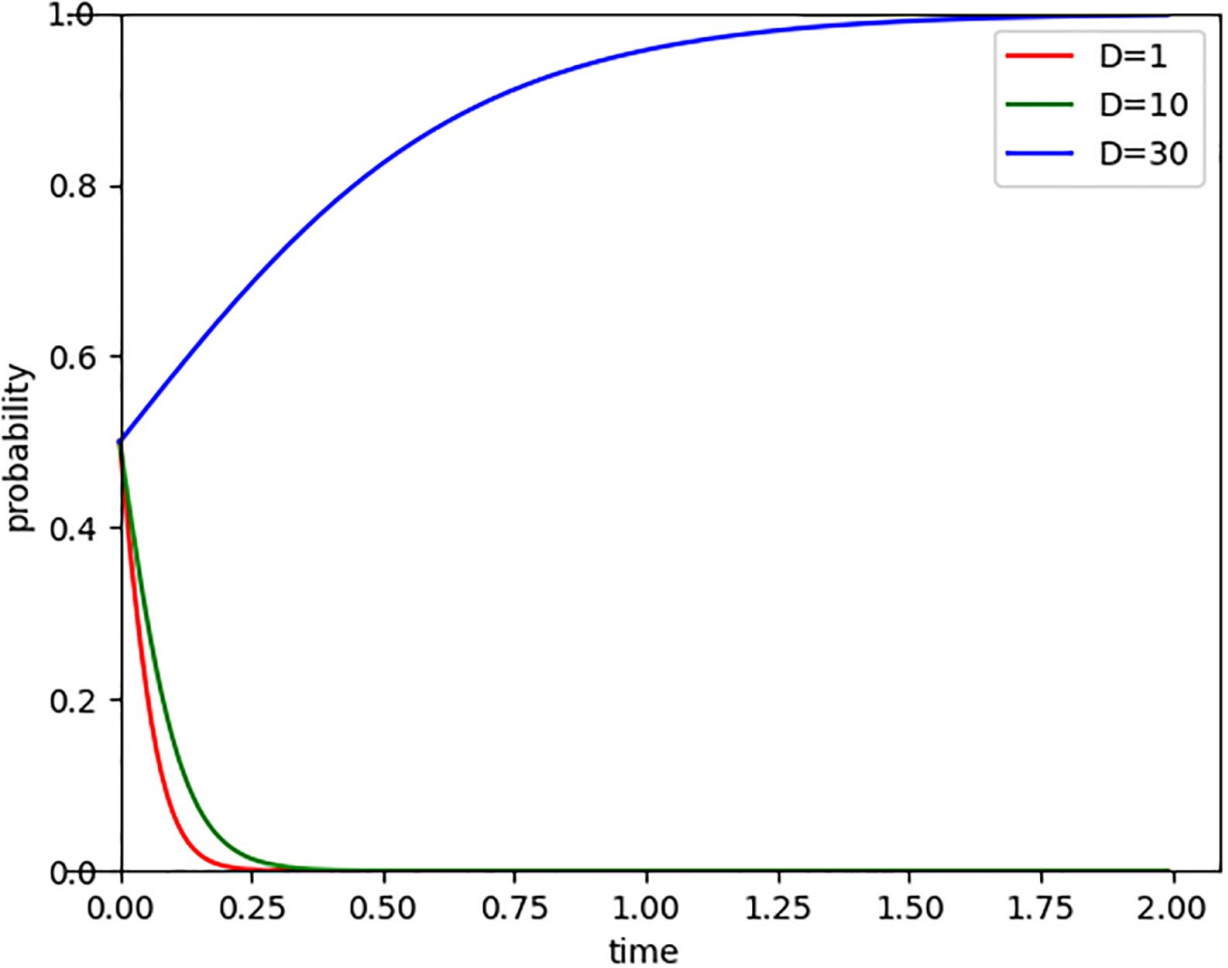
Evolutionary paths of goodwill loss changes.

When a company actively fulfills its environmental responsibilities, such as reducing pollution, decreasing carbon emissions, and promoting sustainable development, it not only enhances its social image and reputation but also gains the trust of the public. Consumers often prefer products and services provided by companies that prioritize environmental protection. Setting goodwill losses (D) at 1, 10, and 30, as shown in Fig 8, the results indicate that as goodwill losses increase, Internet companies’ emphasis on carbon emission reduction and their determination to participate in carbon trading also strengthen. Especially when goodwill losses reach 30, they actively engage in carbon trading. Goodwill, as the economic benefits that a company gains from its good reputation and brand image, appears particularly crucial in modern society. If companies suffer damage due to high carbon emissions or neglect environmental protection, they become aware of the necessity to change their behavior, striving to adopt emission reduction measures, investing in environmental projects, and actively participating in social responsibility activities to repair damaged reputations and rebuild public trust. Therefore, an increase in goodwill losses can encourage Internet companies to pay greater attention to carbon emission reduction and take environmental responsibilities proactively.

## 6. Conclusions and enlightenment

### 6.1 Conclusions

Based on externality and sustainable development theories, this paper constructs an evolutionary game model for Internet companies to enter the carbon market under multiple constraints from the carbon market, financial institutions, and the public. Using Python 3.8.2 software for numerical simulations, we force Internet companies towards low-carbon development and draw the following conclusions:

1. The carbon market can effectively promote carbon emission reduction among Internet companies. The higher the willingness level of Internet companies to enter the carbon market, the longer the evolution time for carbon emission reduction. Coupled with the continuous improvement of the carbon market, the strategy of entering the carbon market gradually becomes the preferred option for Internet companies.
2. The carbon emission reduction behavior of Internet companies has significant externalities. When constraints are not strong or incentives are not obvious, the motivation for carbon emission reduction is insufficient. Because the costs of carbon emission reduction are often borne by the companies themselves, while the environmental benefits brought by emission reduction are shared by the whole society, this asymmetry between costs and benefits leads to a lack of motivation for companies to actively participate in carbon emission reduction without sufficient external incentives.
3. Multiple constraints such as emission reduction costs, penalties for non-compliance, government subsidies, financing costs, opportunity losses, and reputation losses can force Internet companies towards low-carbon development. The carbon market drives companies to reduce emissions by continuously reducing free quotas, thus increasing emission reduction costs. Meanwhile, the carbon market imposes penalties for non-compliance while providing emission reduction subsidies, and strict supervision ensures compliant disclosure and proactive emission reduction by companies. Financial institutions rely on carbon emission reduction support tools to provide financing preferences for low-carbon enterprises, forcing them towards low-carbon development. As public demand for corporate environmental responsibility increases, companies that do not actively reduce emissions face the risk of opportunity losses and reputation losses, compelling them to take proactive emission reduction actions. These multiple constraints accelerate the process of low-carbon development for companies and act as a catalyst forcing Internet companies to reduce emissions.

### 6.2 Management enlightenment

Based on the above analysis, the following management implications can be derived from four perspectives: the government, Internet companies, financial institutions, and the public.

1. Government: The government should strengthen the construction and improvement of the carbon market, increase the willingness level of Internet companies to enter the carbon market, and promote corporate carbon emission reduction. Firstly, it is necessary to improve the policy system of the carbon market, including the carbon emission rights trading system, carbon tax system, etc., to enhance the effectiveness and fairness of the carbon market. Secondly, strengthening carbon market supervision and implementing dynamically adjusted penalty and subsidy measures for non-compliance and emission reduction can encourage companies to comply with disclosures and take proactive emission reduction actions. Thirdly, the government should increase publicity efforts to raise public awareness and emphasis on carbon emission reduction, creating a favorable atmosphere for the whole society to participate in carbon emission reduction.
2. Internet Companies: Internet companies should actively participate in carbon trading, increase their willingness level for carbon emission reduction, strengthen the research and application of low-carbon technologies, and lower emission reduction costs. Firstly, they should develop carbon emission reduction strategies and plans, clarifying emission reduction targets and paths. Secondly, they should enhance technology research and innovation, improving energy efficiency and reducing carbon emission intensity. Thirdly, active participation in carbon market transactions is encouraged, achieving carbon emission reduction through methods such as carbon offsetting and carbon credits. Fourthly, it is important to strengthen the capability of corporate environmental information disclosure, enhancing public awareness and trust in corporate environmental responsibility.
3. Financial Institutions: Financial institutions should rely on carbon emission reduction support tools to provide financing preferences for low-carbon enterprises, promoting low-carbon development. Firstly, they should develop green financial products and services, including green loans, green bonds, green funds, etc., to provide financing support for low-carbon enterprises. Secondly, establishing a green finance evaluation system for credit rating and risk assessment of low-carbon enterprises can enhance the risk management level of financial institutions. Thirdly, strengthening the promotion and advocacy of green finance is crucial to raising public awareness and recognition of green finance.
4. The Public: The public should increase their demands and expectations for corporate environmental responsibility, supervise corporate carbon emission reduction actions, and form a socially co-participatory mechanism for carbon emission reduction. Firstly, raising public awareness and literacy on environmental protection and advocating for green and low-carbon lifestyles and consumption patterns is important. Secondly, establishing a corporate environmental responsibility evaluation system and public supervision mechanism to evaluate and monitor corporate environmental behavior is necessary. Thirdly, strengthening the supervision and reporting efforts of social organizations and the media on corporate environmental responsibility can create societal pressure through public opinion.
